# Facile Synthesis of Mn-Doped ZnO Porous Nanosheets as Anode Materials for Lithium Ion Batteries with a Better Cycle Durability

**DOI:** 10.1186/s11671-015-0983-3

**Published:** 2015-07-03

**Authors:** Linlin Wang, Kaibin Tang, Min Zhang, Jingli Xu

**Affiliations:** School College of Chemistry and Chemical Engineering, Shanghai University of Engineering Science, 333 Longteng Road, Shanghai, 201620 P.R. China; Department of Chemistry, University of Science and Technology of China, Hefei, 230026 P.R. China

**Keywords:** Mn-doped ZnO, Porous nanosheets, Li-ion batteries

## Abstract

Porous Zn_1 − *x*_Mn_*x*_O (*x* = 0.1, 0.2, 0.44) nanosheets were prepared by a low-cost, large-scale production and simple approach, and the applications of these nanosheets as an anode material for Li-ion batteries (LIBs) were explored. Electrochemical measurements showed that the porous Zn_0.8_Mn_0.2_O nanosheets still delivered a stable reversible capacity of 210 mA h g^−1^ at a current rate of 120 mA g^−1^ up to 300 cycles. These results suggest that the facile synthetic method of producing porous Zn_0.8_Mn_0.2_O nanostructure can realize a better cycle durability with stable reversible capacity.

**Electronic supplementary material:**

The online version of this article (doi:10.1186/s11671-015-0983-3) contains supplementary material, which is available to authorized users.

## Background

ZnO, as 3d a transition-metal oxide, has been considered as an anode material for Li-ion batteries (LIBs) due to the following characteristics: high specific capacity (978 mA h g^−1^), abundance, low cost, non-toxic, easily produced, and chemically stable [1–5]. However, it often suffers the loss of capacity upon the cycling due to drastic volume changes during the formation of lithium zinc alloys [6–8]. Therefore, a lot of effort has been devoted to overcome these problems; correspondingly, many methods have been developed, such as (i) preparing ordered nanostructured materials [2, 9]; (ii) incorporating with Ni_3_ZnC_0.7_ [10]; (iii) coating with Ni, C, and CoO-C layers [6, 11, 12]; and (iv) doping with Mg [13]. These techniques enhance the conductivity, facilitate the lithiation/delithiation process, or buffer the volume changes. For example, Ping et al. reported Zn_1 − *x*_Mg_*x*_O (*x* = 0, 0.18) thin films showed an improved cycling stability compared to that of ZnO thin films. The doped Mg ions may only act as a buffer in a form of MgO to alleviate the stress caused by the volume changes during the formation of lithium–zinc alloys [13].

Mn-doped ZnO has been widely investigated for their optical properties, magnetic properties, and sensing properties [14–18]. However, it has rarely been used as anode materials in LIBs. In this work, we successfully synthesized porous Zn_1 − *x*_Mn_*x*_O (*x* = 0.1, 0.2, 0.44) nanosheets in high yield using a facile method. The electrode performance of the samples was electrochemically investigated, and the representative as-synthesized Zn_1 − *x*_Mn_*x*_O (*x* = 0.2) exhibited a better cycle durability with stable reversible capacity of 210 mA h g^−1^ for up to 300 cycles at 120 mA g^−1^.

## Methods

1. ZnO precursor synthesis

The ZnO precursor was prepared by our previous reported method [19]. The ZnCl_2_·5H_2_O (10.0 mmol) and C_6_H_8_O_7_·H_2_O (6.7 mmol) were dissolved in 10-mL water, then solid NaOH (50.0 mmol) was directly added into the mixture solution. Subsequently, 40 mL of distilled water was added and a precursor solution was obtained after 10 min. The white precipitate (flower-like ZnO) was obtained.

2. Synthesis of Zn_1 − *x*_Mn_*x*_O

In a typical process, the obtained ZnO (2.0 mmol) was immersed into Mn(Ac)_2_·4H_2_O (0.2 M, 20 mL) aqueous solution and 40 mL of water was added, stirring for 4 days at room temperature. Then, the resulting powders were obtained and washed with distilled water and ethanol several times, dried at 60 °C. Following, the resulting powders were kept at 350 °C for 3 h under argon atmosphere and the target product Zn_1 − *x*_Mn_*x*_O (*x* = 0.2) was obtained (denoted as sample A). In addition, Zn_1 − *x*_Mn_*x*_O (*x* = 0.1, 0.44) was also obtained with Mn(Ac)_2_·4H_2_O with the amount 8 and 30 mL, respectively (denoted as sample B and sample C).

3. Characterization

The as-prepared particles were characterized by powder X-ray diffraction (XRD) on a Philips X’pert X-ray diffractometer equipped with Cu Kα radiation (*λ* = 1.5418 Å). The morphologies of the samples were examined on a field-emission scanning electron microscopy (FESEM; JEOL JSM-6700 F) and a transmission electron microscopy (TEM; JEOL-2010). The HRTEM images were taken on an aberration-corrected analytical transmission electron microscopy (ARM200F). N_2_ adsorption–desorption isotherms were measured on a Micromeritics ASAP-2000 nitrogen adsorption apparatus at 150 K.

The electrodes for electrochemical testing consisted of 70 wt% active materials, 15 wt% conductive material (acetylene black), and 15 wt% binder (polyvinylidene fluoride (PVDF)). Test cells (2016) were assembled in glove box using lithium metal as the anode, Celgard 2600 as the separator, and 1 M LiPF_6_ in ethylene carbonate and dimethyl carbonate solution (*v*/*v*, 1:1). The galvanostatical charge/discharge measurement was carried out by a LAND-CT2001 battery cycler (Wuhan, China) testing system in the voltage range of 0.01–3.0 V (vs. Li/Li^+^).

## Results and Discussion

**Fig. 1:**
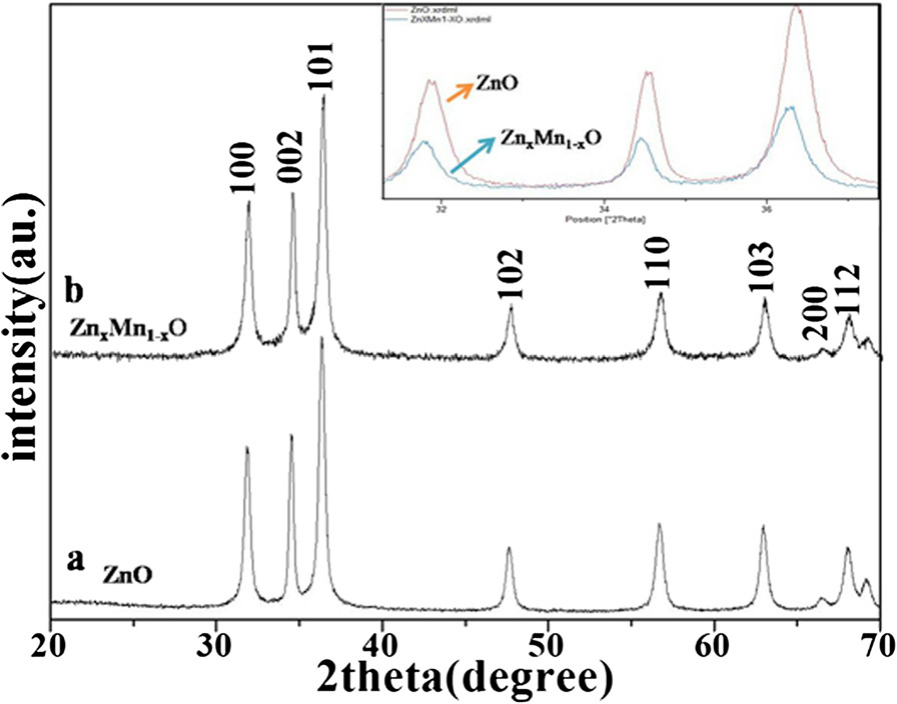
**a** The XRD pattern of the ZnO; **b** the XRD pattern of the sample A and *inset* is an enlarged picture of XRD peaks appearing at ~30°–40°

**Fig. 2:**
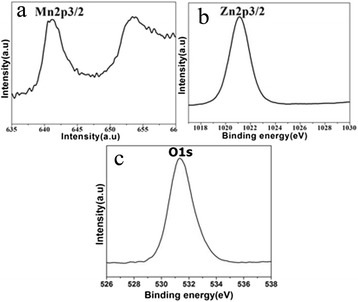
XPS spectra of the sample A: **a** Mn2p3/2, **b** Zn2p3/2, and **c** O1s

**Fig. 3:**
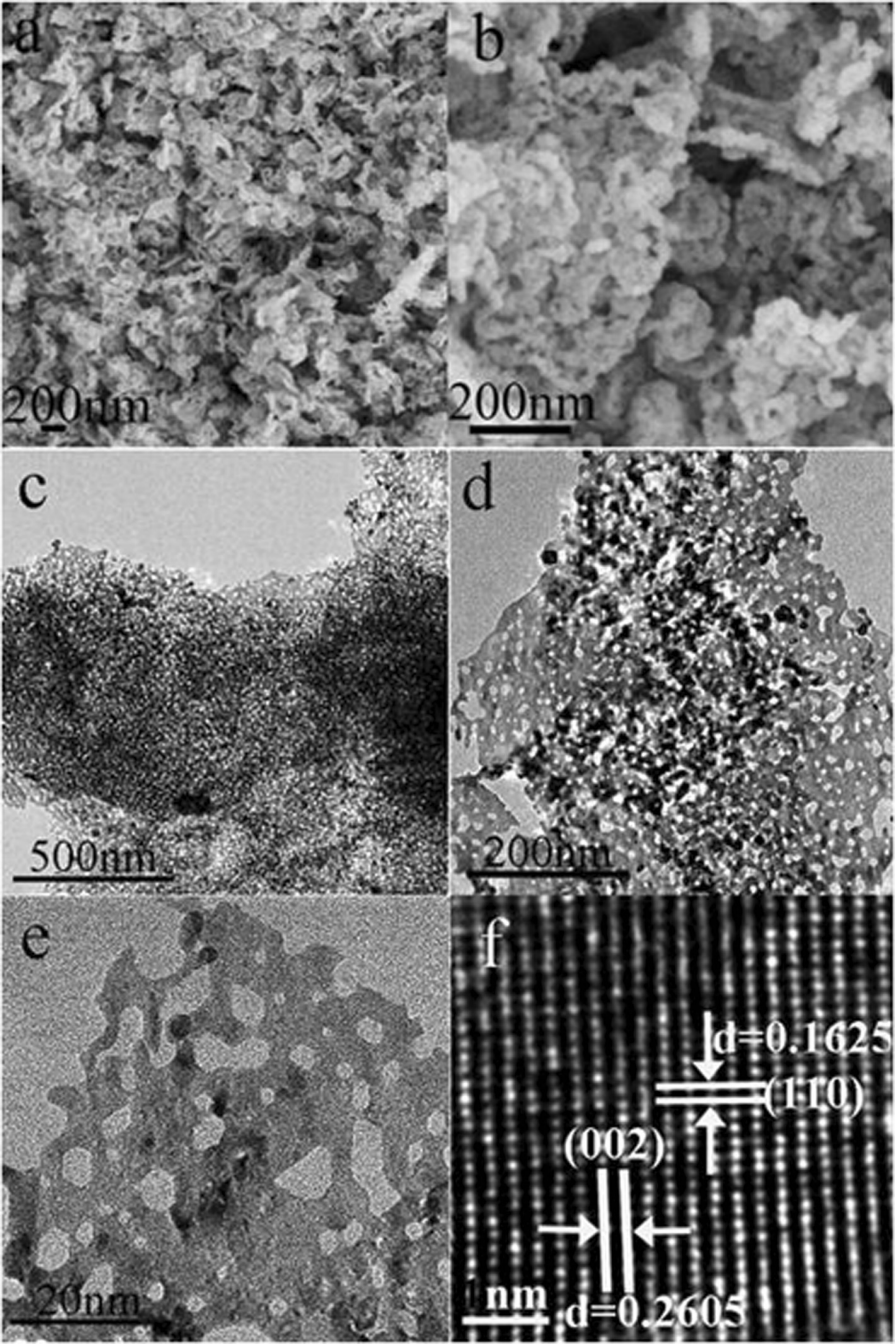
SEM images of the sample A: **a** low magnification, **b** high magnification; TEM images: **c** low magnification and **d**, **e** high magnification; (**f**) a HRTEM image

**Fig. 4:**
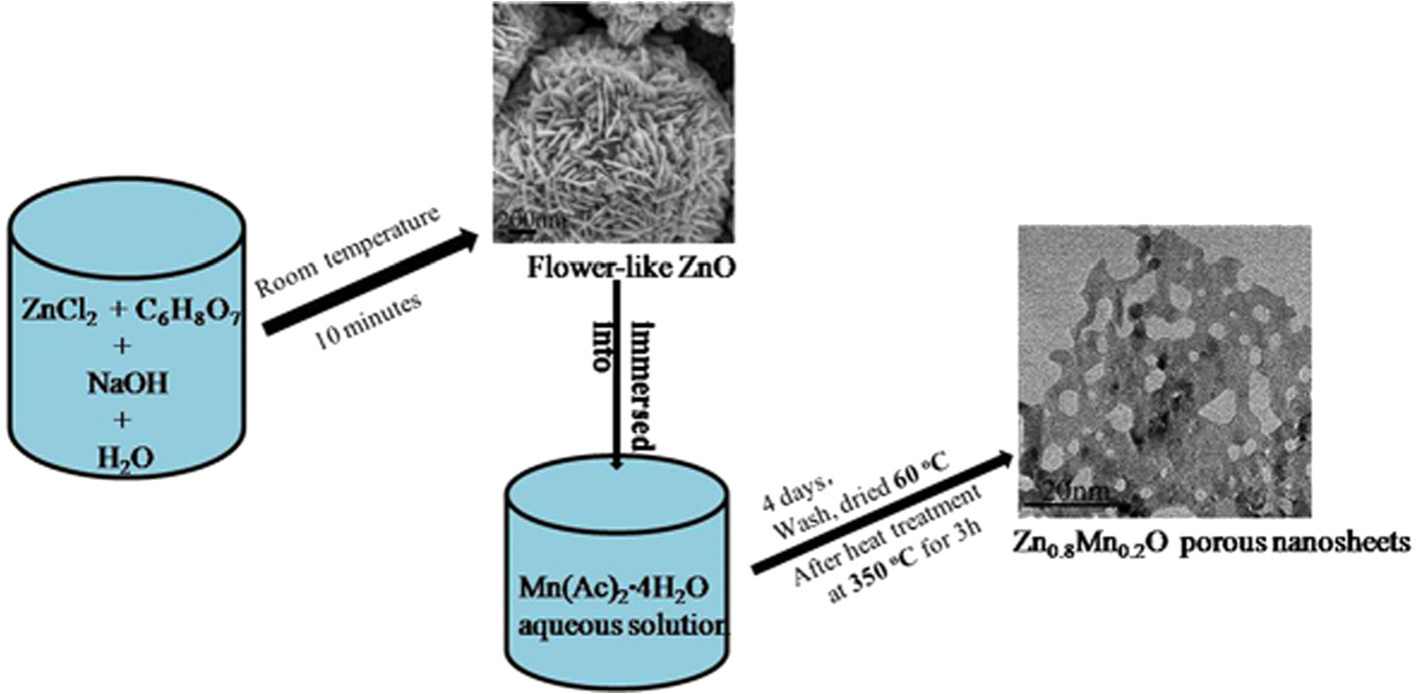
Schematic illustration of the synthesis process of the Zn_1 − *x*_Mn_*x*_O nanosheets

The XRD pattern of the precursor is shown in Fig. 1a. All of the peaks can be assigned to hexagonal ZnO (JCPDS file No. 89-0511). The chemical composition of the sample A was determined by XRD and XPS. The crystallinity and crystal phase of the sample A were demonstrated by the XRD shown in Fig. 1b. The peaks in the diffraction pattern of Zn_1 − *x*_Mn_*x*_O at 31.81°, 34.43°, and 36.30° can be indexed to a hexagonal wurtzite structure, which consists of three prominent peaks corresponding to (100), (002), and (101) planes, respectively. Compared with the peak position of ZnO (inset), that of Zn_1 − *x*_Mn_*x*_O was found to shift towards lower angles with Mn incorporation, probably due to the larger ionic radius of Mn^2+^ (0.066 nm) relative to that of Zn^2+^ (0.060 nm) [20, 21]. The XPS survey spectrum confirms that the sample mainly contains Zn, Mn, and O (Fig. 2). The strong peaks at around 641.7 eV (Fig. 2a) are assigned to Mn2p3/2. The values correspond to a binding energy of Mn^2+^ ion [21]. The peak at 1022.4 eV (Fig. 2c) is assigned to Zn2p3/2 for the Zn^2+^ state (Fig. 2b) and the peak at 531.4 eV (Fig. 2c) corresponds to the binding energy of O1s. In addition, the ratio of Mn to Zn of 6.38:25.05 is given by the quantification of peaks, indicating that the molar ratio of Mn to Zn is near 1:4. The above results indicate that the prepared sample A is single phase Zn_0.8_Mn_0.2_O. The SEM images (Fig. 3a, b) show that the Zn_0.8_Mn_0.2_O samples are sheet-like morphology with many holes in the nanosheets. From the TEM images (Fig. 3c–e), the porous nanosheets were clearly observed, which further confirms the formation of Zn_0.8_Mn_0.2_O porous nanostructures. We also examined the phase purity of the resulting powder that has not been calcined by the XRD. From the XRD pattern (Additional file 1: Figure S1 in supporting information), it is clearly seen that the diffraction pattern of the resulting powder are different from the precursor ZnO and Zn_1 − *x*_Mn_*x*_O. This result suggests that the chemical reaction process (Mn-doped ZnO) is not simple zinc substituted by manganese but involves a complex reaction which led to its structure and composition being changed. The structure and composition of the resulting powder is complex and difficult to identify. However, due to the similar XRD pattern of Zn(OH)_2_ and ZnO, it is reasonable that hydroxide ions might be introduced into the resulting powder during the reaction process, in addition to the possible organic molecules. The morphology of resulting powder (Additional file 1: Figure S2) is nanosheets. Interestingly, after calcination, a number of pores appear on the nanosheets, possibly due to decomposition of the organic molecules and hydroxyl group in the calcination process. The pore size distribution curves have been investigated by using Barrett–Joyner–Halenda (BJH) method. Basing on the report of nitrogen adsorption–desorption shown in Additional file 1: Figure S3, the Zn_0.8_Mn_0.2_O exhibited a BET surface area of 41.45 m^2^ g^−1^ and adsorption average pore diameter of 8.5 nm. For the Zn_0.8_Mn_0.2_O nanosheets, lattice images showed fringes with a spacing of ca. 0.1625 nm and ca. 0.2605 nm, corresponding to the (110) and (002) planes of ZnO (Fig. 3f). An overall schematic model of the synthesis procedure is shown in Fig. 4. The energy dispersion X-ray spectrum (EDS) of the as-prepared sample B and C (Additional file 1: Figure S4) shows that the molar ratio of Mn to Zn of 7.99:66.12 and 16.66:20.96 is near 1:8 (Zn_0.9_Mn_0.1_O) and 1:1.25 (Zn_0.56_Mn_0.44_O), respectively.

**Fig. 5:**
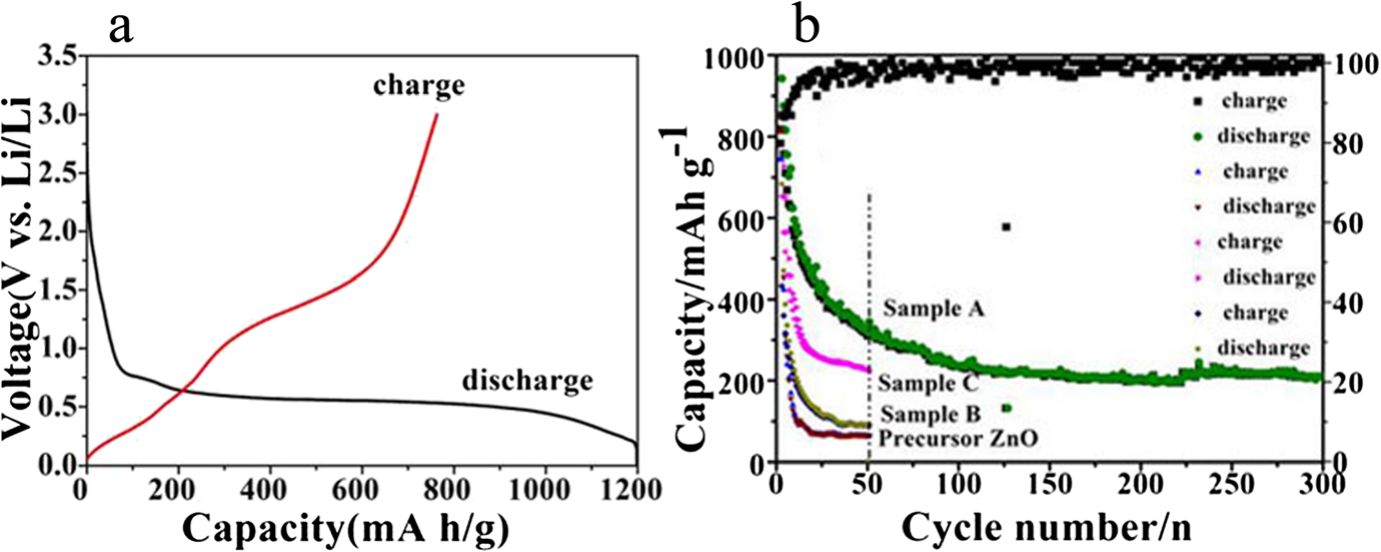
**a** Initial discharge/charge profile for the Zn_0.8_Mn_0.2_O; **b** cycling performances and Coulombic efficiencies of the Zn_1 − *x*_Mn_*x*_O and ZnO electrodes

The electrochemical properties of the porous Zn_0.8_Mn_0.2_O nanosheets (sample A) were characterized by a galvanostatic method. Figure 5a, b shows first charge/discharge profiles and cycling performance of the Zn_0.8_Mn_0.2_O electrode tested in the potential range of 0.01 to 3.0 V and at a current density of 120 mA h g^−1^. The Zn_0.8_Mn_0.2_O nanosheets exhibit the initial discharge and charge capacities of 1198 and 763 mA h g^−1^, with an initial coulombic efficiency of 64 %. After 50 cycles, this Zn_0.8_Mn_0.2_O electrode delivers a reversible capacity of 340 mA h g^−1^. Even at 300 cycles, this Zn_0.8_Mn_0.2_O material still retains a stable capacity of 210 mA h g^−1^, thus exhibiting an excellent cycle durability. For comparison purposes, we also investigate the electrochemical properties of sample B, sample C, and the ZnO precursor (Fig. 5b). Sample B retains a reversible capacity of ~220 mA h g^−1^ up to 50 cycle, and the sample delivers a lower capacity of ~90 mA h g^−1^, while the capacity of the ZnO deteriorates severely and ZnO delivers a much lower capacity of 64 mA h g^−1^ up to 50 cycles. Apparently, the reversible capacity of the Zn_0.8_Mn_0.2_O porous nanosheets is enhanced than that of sample B, sample C, and the precursor ZnO. The good cycle durability of the Zn_0.8_Mn_0.2_O material might be reasonable to be attributed to the fact that a great number of the cavities among nanosheets are all beneficial to relieve the strain induced by the severe volume variations of Zn_0.8_Mn_0.2_O during Li^+^ uptake–release, which might improve better rate capacity and the cycling performance.

## Conclusions

In conclusion, the Mn-doped ZnO porous nanosheets were successfully synthesized by a simple approach and their electrochemical performance were evaluated. The obtained Zn_0.8_Mn_0.2_O porous nanosheets exhibit better cycle durability with good reversible capacity. The cavities among nanosheets maybe could effectively suppress the volume expansion during cycling and enhances the electric conductivity of electrodes, etc., giving rise to better electrochemical performance and cycling stability. In addition, our results provide a simple, effective strategy to fabricate the Zn_0.8_Mn_0.2_O nanostructure.

## Additional File

Additional file 1:
**The XRD pattern of three samples, SEM image of the resulting power, Nitrogen adsorption/desorption isotherms and pore size distribution of Zn**_**0.8**_ **Mn**_**0.2**_ **O and EDS spectrum of the products. Figure S1.** XRD pattern of three samples, **Figure S2.** SEM image of the resulting power, **Figure S3.** Nitrogen adsorption/desorption isotherms and pore size distribution (inset) of Zn_0.8_Mn_0.2_O (sample A). **Figure S4.** EDS spectrum of the products (a) sample B; (b) sample C.
