# Supplementary material for: Facile Synthesis of Mn-Doped ZnO Porous Nanosheets as Anode Materials for Lithium Ion Batteries with a Better Cycle Durability
Source: Nanoscale Res Lett. 2015 Jul 3;10:280. doi: 10.1186/s11671-015-0983-3 (PMC4489971; doi:10.1186/s11671-015-0983-3)
Supplement: Additional file 1: — The XRD pattern of three samples, SEM image of the resulting power, Nitrogen adsorption/desorption isotherms and pore size distribution of Zn 0.8 Mn 0.2 O and EDS spectrum of the products. Figure S1. XRD pattern of three samples, Figure S2. SEM image of the resulting power, Figure S3. Nitrogen adsorption/desorption isotherms and pore size distribution (inset) of Zn0.8Mn0.2O (sample A). Figure S4. EDS spectrum of the products (a) sample B; (b) sample C. [file 11671_2015_983_MOESM1_ESM.docx]

**Facile synthesis of Mn-doped ZnO porous nanosheets as anode** **materials for lithium ion batteries with a better cycle durability**

Linlin Wang,^1,2^ Kaibin Tang,^2 *^ Min Zhang^1^ and Jingli Xu^1*^

^
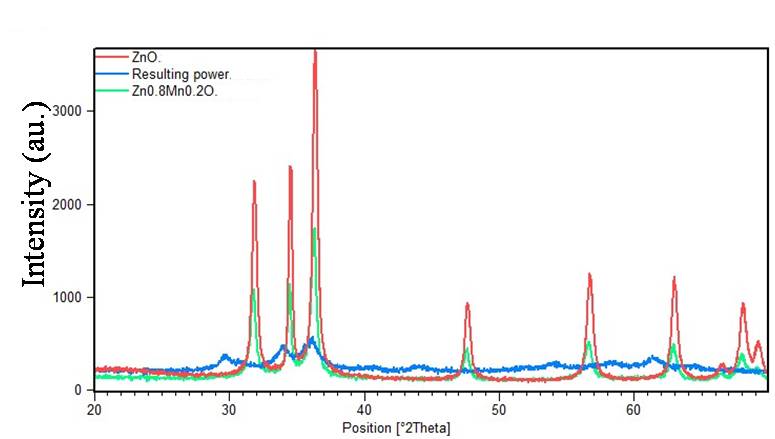
^

Figure S1 XRD pattern of three samples





Figure S2 SEM image of the resulting power


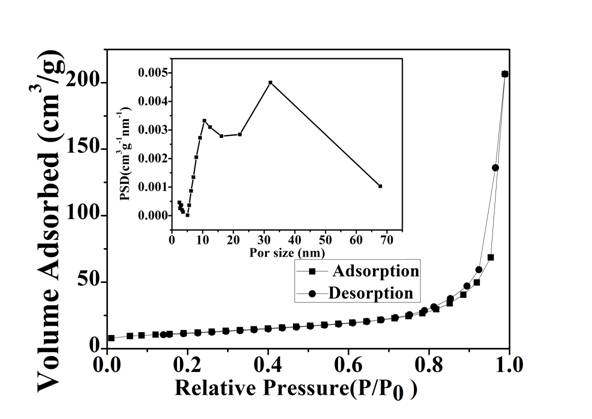


Figure S3 Nitrogen adsorption/desorption isotherms and pore size distribution (inset) of Zn_0.8_Mn_0.2_O (sample A).


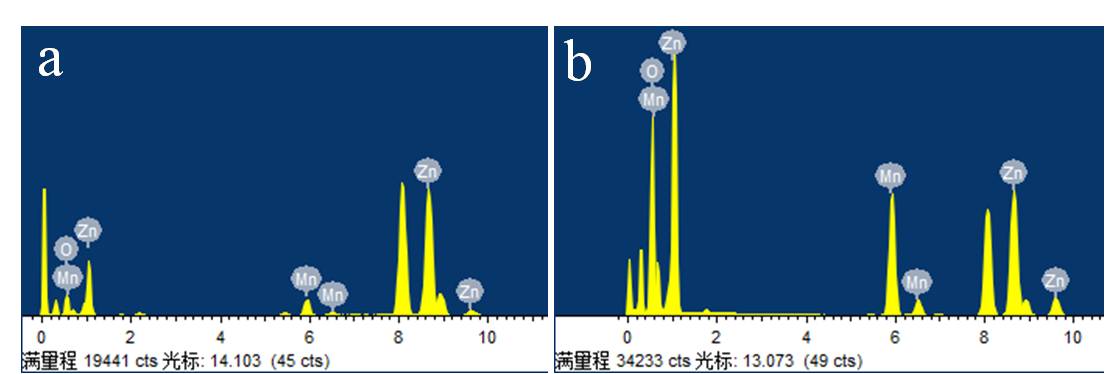


Figure S4 EDS spectrum of the products (a) sample B; (b) sample C
